# Alberta Rating Index for Apps: Study of Reliability and Validity

**DOI:** 10.1177/00084174221085451

**Published:** 2022-03-16

**Authors:** Peyman Azad-Khaneghah, Mary Roduta Roberts, Lili Liu

**Keywords:** Health care, Mental health, Mobile applications, Quality assurance, Telemedicine, Applications mobiles, assurance de la qualité, santé mentale, soins de santé, télémédecine

## Abstract

**Background.** The number of mobile health applications is rapidly increasing, yet no reliable tool exists for occupational therapists and their clients to rate the quality of these apps. **Purpose**. To develop the Alberta Rating Index for Apps (ARIA). **Methods.** Through a sequential design in three phases, we developed a rating index for mobile health applications and examined its reliability and validity with 10 participants. **Findings.** The coefficients of reliability were 0.95 for occupational therapists, 0.60 for older adults, and 0.88 for adults with a mental health condition. ARIA's correlation with another scale used in app review studies, U-MARS, was low to moderate. **Implications.** ARIA showed a high inter-rater reliability in two of the three user groups. ARIA is comprehensive and includes criteria not captured by U-MARS, such as privacy and security. Further studies are warranted with diverse raters and health apps.

## Résumé

**Description.** Le nombre d’applications de santé mobiles augmente rapidement, mais il n’existe pas d’outil fiable permettant aux ergothérapeutes et à leur clientèle d’en évaluer la qualité. **But.** Élaborer le Alberta Rating Index for Apps (ARIA). **Méthodologie.** Par l’intermédiaire d’une étude séquentielle en trois phases, nous avons élaboré un indice d’évaluation des applications de santé mobiles et examiné sa fiabilité et sa validité auprès de 10 participants. **Résultats.** Le coefficient de fiabilité était de 0,95 pour les ergothérapeutes, de 0,60 pour les personnes âgées et de 0,88 pour les adultes avec un problème de santé mentale. La corrélation de l’ARIA avec une autre échelle utilisée pour évaluer les applications, U-MARS, était de faible à modérée. **Conséquences.** L’ARIA a démontré une fidélité interjuges élevée dans deux des trois groupes d’utilisateurs. L’ARIA est exhaustif et comprend des critères non pris en compte par U-MARS, comme la confidentialité et la sécurité. Des études supplémentaires s’imposent, avec une diversité d’utilisateurs et d’applications.

## Introduction

Mobile health applications (mhealth apps) are software that run on mobile devices, such as smartphones and tablets (Lui et al., 2017). In recent years, the professional occupational therapy associations in North America have advocated for occupational therapists to use technologies such as mhealth apps in their practice (Ravenek & Alvarez, 2019). Clients and their family caregivers commonly consult occupational therapists to recommend or advise on a “*good mobile app*” for themselves or their loved ones under their care. As part of their professional role as patient educators and advocators (Townsend & Polatajko, 2007), occupational therapists benefit from “approaches that enhance their clinical reasoning as it pertains to the evaluation, critical appraisal and use of apps for occupational therapy interventions” (Ravenek & Alvarez, 2019, p. 43). This paper introduces a new tool that can help occupational therapists with this task.

More than 300,000 mhealth apps exist (Research2Guidance, 2017), and over 10,000 of these are related to mental health conditions (Torous & Roberts, 2017). Mobile health apps for mental health aim to improve mental health and well-being; these range from helping with mental illness recovery to maintaining habits that contribute to emotional health (Bakker et al., 2016). Studies show that users are attracted to mental health apps based on their accessibility, immediacy, affordability, or sometimes bold marketing claims (Torous & Roberts, 2017). There is increasing interest in using mobile phones for mental health monitoring and self-management (Donker et al., 2013).

The escalating number of mhealth apps along with increasing interest among patients raise concerns among some health practitioners, including occupational therapists. These concerns are related to the apps’ effectiveness (Larsen et al., 2016), risk of inaccurate or harmful information, and the apps’ ability to protect users’ information (BinDhim et al., 2014). Nevertheless, studies have also reported some apps that are safe, evidence-based, and useful (Lui et al., 2017). It can be a challenge for consumers and health practitioners to distinguish between apps that are safe to use from ones to avoid (Torous & Roberts, 2017).

Regulations by the United States Food and Drug Administration (2019) and Health Canada (2018) may not apply to apps related to mental health such as mood journals, relaxation apps, or apps used for cognitive behavioural therapy. In the absence of regulations and strict quality measures for mhealth apps, consumers rely on other ways (Leigh, 2016) such as app descriptions on app stores and ratings or reviews from other users on the internet and social media (Google, 2018). However, these ratings and reviews may be unrealistic, make exaggerated claims, be inaccurate, or not relevant to all consumers (BinDhim et al., 2015; Singh et al., 2016).

Recently, professional organizations have reviewed health apps and published the results in the form of app directories or online app libraries. Examples include the United Kingdom's National Health Services app library (2017), and the Alberta Health Services Addiction and Mental Health Mobile Application Directory (2018). The app libraries may be perceived as reliable sources of information by patients and clinicians. However, they are not comprehensive and include only a portion of mhealth apps available on app stores during a specified period. Due to the short lifecycle of mhealth apps, app directories require frequent updates to be useful which consumes considerable resources.

Recent initiatives include developing smart programs that assist users with searching a library of pre-screened apps and selecting the ones that match their needs. An example is Find My Apps programme (Beentjes et al., 2020). Despite their potential in certain circumstances, they share the same shortcomings as app directories. Moreover, such programs are only applicable to a certain number of apps and cannot help users navigate the uncharted territory of online app stores.

An alternative approach is to enable consumers and health practitioners to evaluate the quality of apps using a criteria-based rating scale. A systematic review of the literature indicates at least 25 different app rating scales exist (Azad-Khaneghah et al., 2021). However, current mobile app rating scales poorly describe the connection between the criteria measured with the quality of mobile health apps. Some exclude essential criteria. For example, the Mobile Applications Rating Scale (MARS) (Stoyanov et al., 2015) is a relatively new scale that has been widely used in several app review studies (e.g., Adam et al., 2018; Bardus et al., 2016; Grainger et al., 2017; Mani et al., 2015; O'Reilly et al., 2018). MARS measures user engagement, functionality, aesthetics, and quality of information in apps, but it overlooks privacy and security. There is also diversity and no standardization in essential criteria for the evaluation of quality of mobile health apps. The selection of quality criteria forming the basis of current scales is arbitrary, at the discretion of the authors, and not guided by theoretical frameworks (Azad-Khaneghah et al., 2021).

Almost all existing scales were developed for health care professionals or researchers (Azad-Khaneghah et al., 2021). Members of the lay population were not involved in the development process of any of these scales. The number of older adults, aged 65 years or older, using information communication technology for health is increasing (Anderson, 2017; Smith, 2014), however, their needs were overlooked in most of these scales (Singh & Bajorek, 2014). Older adults are more likely to face barriers when using smartphones due to age-related decline in sensory, motor, and cognitive skills. Small screen sizes of smartphones combined with touch screen navigation and complex human-interface interactions (e.g., pinch, swipe, or double tap) can be challenging for adults with age-related perceptual and sensory changes (Wildenbos et al., 2015).

The objective of this study was to develop the Alberta Rating Index for Apps, or ARIA, a rating index supported by technology acceptance models and frameworks of app evaluation, which is usable by younger and older adult consumers of mental health mobile applications as well as their family caregivers and health care providers.

## Method

The study received ethics approval from the Human Research Ethics Board of the University of Alberta on February 9, 2017, protocol number Pro00067856.

We developed ARIA using a sequential design in three phases, each phase conducted in two steps. The results of each step informed specifics of the implementation of the following step (Streiner et al., 2015). In step one of phase one, we determined the quality criteria that are relevant to mhealth apps using focus groups. In step two of phase one, we developed an item pool to assess these criteria. We followed the methodology used by Nouri et al. (2018) to identify and categorize items under different quality criteria. Following a systematic review of the literature (Azad-Khaneghah et al., 2021), we identified 25 rating scales used to rate the quality of online health interventions. The first author extracted items from these scales, compared them, and grouped similar ones under relevant quality criteria that were identified in step one. A panel of three researchers (the second author and two arms length researchers) with experience in evaluation of health technology and scale development, reviewed these items and determined their relevance to the given criteria. We excluded redundant items and items that were deemed irrelevant by at least two of the panel members. We edited and revised the remaining items to improve readability. The outcome was an initial pool of 74 items.

In step one of phase two, we validated the items in the item pool using an online survey (details are in Table 1). With assistance from the Mental Health Commission of Canada, we shared the link of the online survey with a network of their research collaborators. The network included individuals with lived experience of a mental health condition, older adults, app developers, and mental healthcare providers who participated in the survey anonymously. We requested participants to rate the relevance of each item from the item pool to the given criterion using a three-point scale (0 = not relevant at all, 1 = relevant but needs revision, and 2 = relevant as is). Next, we calculated a content validity index following Lynn's method (1986). Based on Lynn's criteria, items endorsed by at least 80% of the participants as relevant were considered valid. Other items were excluded. The outcome was a pool of 53 items.

**Table 1:** Summary of Previous Phases of the Study

Study phase	Objective	Data collection method	Participants^a^	Procedure	Outcome
1	Identify quality criteria relevant to mhealth apps	Focus groups conducted in-person	Convenience sampling Older adults (n = 7) recruited from Seniors Association of Greater Edmonton, Alberta Adults living with a mental health condition (n = 7) recruited from Organization for Bipolar and Affective Disorders (OBAD) Calgary, Alberta Mental health care providers (n = 11) recruited from Department of Occupational therapy, University of Alberta and practitioners at a mental health clinic in Clagary, Alberta App developers (n = 7) recruited from Department of Computing Science, University of Alberta	Total of six homogenous focus groups were conducted: two focus groups for older adults, one for app developers, one for adults with mental health condition, and two for healthcare providers. Participants reviewed 11 criteria from the following models and frameworks: Unified Theory of Acceptance and Use of Technology (UTAUT) (Venkatesh et al., 2012) Seniors Technology Acceptance Model (STAM) (Renaud & Biljon, 2008) Nielsen Heuristics of Usability (Nielsen, 1993) Health on the Net foundation (HON) code (HON, 2019) App Synopsis (Albrecht, von Jan, Jungnickel, & Pramann, 2013) Mental Health Commission of Canada's framework of App evaluation (Zelmer et al., 2018)	9 criteria were validated as relevant to mhealth apps: purpose of the app, trustworthiness, compliance with privacy policies, security of the data, affordability, ease of use, functionality, appropriate to the target users, usefulness, users’ satisfaction
	Develop an item pool	Review of the literature and mapping	Convenience sampling Panel of reviewers (n = 3) recruited from Department of Occupational therapy, University of Alberta	Scales and assessment tools for rating mobile health apps were identified in a systematic review (Azad-Khaneghah et al. 2021) Items were extracted and mapped to the criteria validated in previous step Redundant items were deleted, and similar items were merged, items were rephrased to better measure the criteria	Developed 74 items unevenly distributed across 9 quality criteria after reviewing mobile app rating scales
2	Content validation	Online survey using Survey Gizmo (now named Alchemer, 2020)	Participants were recruited with assistance of our collaborator at Mental Health Commission of Canada (MHCC). Link of survey was circulated with MHCC network of collaborators. We received 18 responses from the following: Mental health care providers (n = 8) Adults living with a mental health condition (n = 6) Older adults (n = 1) App developers (n = 3)	Items were reviewed for relevance to the given criteria Relevance rated using the following scale: 0 = not relevant at all, 1 = relevant but needs revision, and 2 = relevant as is Calculated content validity index (CVI) following Lynn (1986) criteria.	53 items were determined as valid (CVI≥0.8) Additional items were suggested by the participants
	Item reduction	Focus group conducted in-person	Adult living with mental health condition (n = 1) Usability researcher (n = 1) Mental health care providers (n = 4) Older adults (n = 2) Participants were recruited from phase one of the study	Participants reviewed the items two weeks in advance of the focus group. On the day of the focus group, they discussed the items and voted which ones are relevant to the index and which ones are not.	Reduced the number of items to 18. Pilot version of Alberta Rating Index for Apps (ARIA) was developed
3	Pilot testing	Think out loud method	Adults living with a mental health condition (n = 7) Older adults (n = 2)	Conducted in person with researcher present. Participants applied ARIA to two apps selected from Alberta Health Service's Addiction and Mental Health Mobile App Directory	Readability and comprehensibility of the items were tested Two versions of ARIA were developed: Care providers version and users’ version Scaling anchors were reworded to resemble traditional Likert scales (0 = completely disagree, 1 = Disagree, 2 = Neutral, 3 = Agree, 4 = Completely agree)
	Reliability and validity study	Reliability testing using 11 mental health mobile apps Validity testing against Users Version of Mobile Application Rating Scale (UMARS)	Mental health care providers (n = 4) Older adults (n = 3) Adults living with a mental health condition (n = 3)	Participants rated 11 apps, using ARIA and U-MARS, at their home.	Reliability is moderate to high ARIA correlates moderately with U-MARS

^a^ All participants were recruited using a convenience sampling method and provided informed consent. Participants in "item reduction study" also participated in phase one focus groups. From all raters in phase three, four adults with mental health condition, two older adults, and two mental health care providers were also involved in phase one focus groups. All other participants were only involved in one study.

In step two of phase two, we reduced the number of items using an in person focus group (details are in Table 1). This step resulted in the first version of ARIA. In step one of phase three, we pilot tested the items in the first version of ARIA. Finally, in step two of phase three, we tested the reliability and validity of ARIA (Table 1). Throughout the development phases, we followed the universal design principles (1997), specifically flexibility in use, simple and intuitive use, and low physical effort.

This paper presents the details of step two of phase three, the reliability and validity testing of ARIA. We determined the inter-rater reliability of ARIA using a fully crossed design within the framework of generalizability theory (Brennan, 2001). In this design, multiple raters were recruited from different groups of potential users of mhealth apps. Each participant used ARIA to rate all apps selected for this study (raters × apps). The fully crossed design enabled us to investigate the different sources (i.e., raters, apps) that could contribute to score variability and estimate reliability. Since the index was a rating-based instrument and in the development phase, it was more important to investigate the extent to which the total score captured the differences in app quality and variability rather than the agreement between raters, when estimating reliability. Hence, a generalizability study served our purpose better than other analyses such as intra-class correlation coefficient.

To investigate the construct validity of ARIA, the average total ratings of ARIA and Users version of Mobile Application Rating Scale (U-MARS) (Stoyanov et al., 2016) were calculated for the same apps used by the same group of raters and then correlated. Currently no gold standard rating scales exist for mobile health apps. U-MARS is the user version of MARS, which is commonly used in app review studies ARIA.

### Participants

We used a convenience sampling method (Portney & Watkins, 2009) to recruit for participants of this study (hereafter referred to as raters). All participants met the following general inclusion criteria:

- At least 18 years,
- Able to communicate in English (verbal and written),
- Owned and used a smartphone for at least one year.

At least two raters were needed to estimate the rater variance component using generalizability theory. We examined the reliability of scores within three user groups: adults with a mental health condition (i.e., mood disorder), health practitioners (here, occupational therapists), and healthy older adults (aged ≥65). Also, to investigate any effects of the operating system used, for each group we recruited two participants who used an iPhone and two who used an Android phone. As a result, we recruited four participants for each group (10 completed the study).

We recruited four occupational therapists from faculty members, clinical instructors, and occupational therapist researchers who worked at the Department of Occupational Therapy at the University of Alberta. The first author sent an invitation email to the potential participants who met the inclusion criteria and invited them to the study. The first four respondents who met the inclusion criteria were recruited. Two of them had also participated in phase one of the study.

Four adults living with a mental health condition were recruited as the main target group of the index since they may most likely use mental health mobile apps. Participants were recruited from Organizations for Bipolar and Affective Disorders (OBAD) in Calgary, Alberta, and had previously participated in phase one of the study. The members of OBAD are individuals who have a history of a chronic bipolar or affective disorder. The first author sent the information letter and invitation email to the OBAD administrators, who then forwarded the study information to their members. Individuals who were interested contacted the first author by email or phone.

In addition, we recruited four older adults who self-identified as healthy. This group was recruited for their unique experience with age related limitations in the use of mobile health apps. Moreover, mental health mobile apps can be used for both prevention and treatment of mental health conditions. Many older adults are at risk of mental health conditions such as depression and anxiety as they age (Butler et al., 1997) that may go undetected (Padayachey et al., 2017). Participants in this group were recruited from individuals who collaborated with our research team in previous projects. We sent the information letter to them by email and invited them to participate in the study. Those who were interested contacted the first author. We recruited four volunteers who met the inclusion criteria, two of whom had participated in phase one of the study as well.

For all groups, individuals were excluded if they:

- Had severe physical, visual, and hearing limitations that could not be accommodated by using an assistive device
- Were unable to provide informed consent

### The index

Alberta Rating Index for Apps, or ARIA (Supplemental Appendix 2), is an index with 18 items, in two versions: one for those who are interested in rating the quality of health apps for their own use (the user version) and one for those such as healthcare providers or family caregivers, who are interested in rating the quality of health apps as a proxy for a person under their care (the care provider version).

The index items are composed as statements about different quality criteria that pertain to mobile health apps (e.g., purpose, privacy, usefulness, and ease of use). Raters express their agreement with each item using a Likert scale from 0 to 4 (0 = Strongly disagree, and 4 = Strongly agree). The mid-point was “Neutral.” The index has two parts. Part A contains six items that help users pre-screen an app and decide if they may consider downloading the app. Part B includes 12 items that are designed to help users determine the usability and quality of the content of an app after they have decided to download it. To calculate the index score, raters add the scores given to each item. Part A scores range from 0 to 24, and Part B scores range from 0 to 48 with higher scores corresponding to better app quality.

### M-Health Apps

The search feature of app stores is currently designed to show apps based on popularity and download rates. This may not necessarily retrieve a diverse sample of apps or the most appropriate apps for users. Instead, we selected apps from the Alberta Health Services Addiction and Mental Health Mobile Application Directory (2018). The directory was developed through a comprehensive search for apps in different information sources in the public domain. It includes apps for various mental health conditions. However, Alberta Health Services does not evaluate the quality of the apps in this directory although they are from trackable developers.

The 2018 directory included 39 apps under several categories of mental health conditions. We pre-screened and shortlisted the apps on the directory based on the following criteria:

- Available on both iOS and Android markets,
- Developed for users in the general population with no medical training,
- Designed for mental health conditions common in adults and older adults.

Fifteen apps were shortlisted that met the above criteria. We randomly selected one app from categories that had two apps, and two apps from those that contained three apps. We picked both apps in the mood disorder category for their relevance to a group of our participants. The result was as follows:

- Substance use disorders (2 apps)
- Mood disorders (2 apps)
- Anxiety disorders (1 app)
- Post-traumatic stress disorder (PTSD) (1 app)
- Eating disorder (1 app)
- Stress management (2 apps)
- Medication management (1 apps)
- Sleep management (1 app)

In addition to the above 11 apps used for the reliability study, the first author randomly selected two apps from the remaining four not already selected and used them for training the participants.

### Procedure

All raters provided informed signed consent. Next, the first author demonstrated to each rater how to use ARIA and U-MARS. Each rater applied ARIA and U-MARS in a practice session on the same two practice apps (selected from the directory as described above) in the presence of the first author. Raters had the opportunity to ask questions until they felt comfortable using both ARIA and U-MARS.

Each rater received the list of 11 apps and adequate paper copies of ARIA and U-MARS to take home. Raters were instructed to download all 11 apps on their smartphone and use the apps for as long as they needed to become familiar with the apps’ features. This time varied for different apps.

We instructed the raters in the occupational therapist group to use the care provider version of ARIA and rate the apps, as if they wanted to recommend an app to a mock client (specifics in Supplemental Appendix 3). Raters in the older adults and adults with a mental health condition groups were instructed to use the user version of ARIA and rate the apps considering their own preferences, needs, and expectations (i.e., as if they wanted to use the apps for themselves).

The raters were asked to complete their ratings of all 11 apps within a 30-day period. During this time, they could contact the first author if they had any questions. The first author also phoned each rater once a week to follow up. After 30 days, the first author met with each rater and collected completed scales. A semi-structured short interview was conducted with each rater in person to record their feedback about using ARIA.

### Data Analysis

We conducted a single-facet generalizability study (G-study) (Brennan, 2001) with quality of apps fully crossed with raters (i.e., App × R) for each rater group independently (only within group analysis was conducted). We investigated the proportion of variance associated with quality of apps and raters in each group, as well as the G-coefficient as the measure of inter-rater reliability for the total index score (Nunnally, 1967). To test the criterion-related validity of ARIA, we correlated the total scores from ARIA in each group with the total scores from U-MARS for the same apps and the same group of raters. Raters’ comments from the interview were recorded digitally and transcribed verbatim and analyzed using a thematic analysis method as described by Braun and Clarke (2006).

IBM SPSS Statistics for Windows (Version 25.0) (2017)was used for all the statistical analyses. A peer-reviewed SPSS Syntax (Mushquash & O’Connor, 2006) was used to estimate the variance components and the G-coefficient in the G-Study. The type one error (alpha) was set at 0.05 for all analyses.

## Findings

### Participants’ Characteristics

We started the study with 12 raters from three different groups of stakeholders. They used the version of ARIA appropriate to their cohort to rate the quality of 11 mhealth apps related to mental health. One rater from the older adults’ group and one from the adults with mental health condition group did not complete the study due to personal reasons not shared with the investigator. Hence, we completed the final analysis with 10 raters (Table 2). No participant reported any adverse effects such as worsening of symptoms (i.e., in adults with a mental health condition) or experiencing mental fatigue.

**Table 2:** Participants’ Characteristics

Participant group	Gender (female/male)	Mean age (SD)	Phone used (iPhone/Android)
Occupational therapists	4/0	35 (8.3)	2/2
Older adults	0/3	68.6 (9)	2/1
Adults with mental health condition	1/2	47.6 (10.9)	2/1

### App Ratings by Group

**Figure 1.**
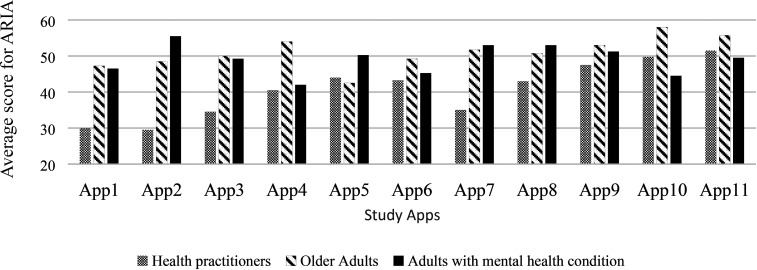
Distribution of average total ARIA score for each app across each group of raters.

The average total scores for each app were different between groups (Figure 1). To prevent any inadvertent promotion of specific apps used in this study, we blinded the names of the apps in this figure. Mixed-factorial analysis of variance (ANOVA) confirmed a significant between-group difference in ratings (*F* (2, 30) = 10.45, *p* < .01) with the occupational therapist group scoring the apps significantly lower than the older adult group (mean difference = 10.2, 95% CIs [16.2, 4.2]) and the adults with mental health condition group (mean difference = 8.3, 95% CI [14.3, 2.3]).

We compared the average total scores between the iPhone users and Android users within each group using a one-way ANOVA to investigate any possible impact of the operating system used by the participants on their ratings. No significant differences were found between total scores of iPhone users and Android users for the same app in any of the rater groups.

### Inter-Rater Reliability

A single facet G-study was conducted on apps and raters (App × R), using the total score of ARIA for each rater group separately. The proportion of variance associated with apps (APP), raters (R), and error interaction (APP × R, e) for each group are presented in Table 3.

**Table 3:** Variance Decomposition Table (Analysis in Each Group is Independent of the Others)

Group	Source	df	Variance	Proportion of variance explained	*G*
**Occupational therapists**	**APP**	10	54.808	0.816	0.95
	**R**	3	0.226	0.003	
	**APP × R, e**	30	12.092	0.18	
**Adults with mental health condition**	**APP**	10	26.915	0.656	0.88
	**R**	2	2.648	0.065	
	**APP × R, e**	20	11.442	0.279	
**Older adults**	**APP**	30	45.953	0.547	0.60
	**R**	10	21.758	0.29	
	**APP × R, e**	2	11.23	0.15	

APP = Quality of apps (object of measurement); R = Raters (Facet 1), e = error, G = coefficient of reliability.

It is recommended that 0.8 be the minimal cut-off for acceptable reliability with instruments used for research applications and program evaluations (Nunnally, 1967). Also, in the context of generalizability theory, it is desirable for the proportion of variance associated with the target or object of measurement (i.e., quality of the apps) to be higher relative to the proportion of variance associated with raters or error. The reliability coefficient was highest in the occupational therapists’ group (*G* = 0.95). Correspondingly, the largest proportion of observed variance was attributable to differences between app quality (81.6%) which is a desirable outcome. The variance proportion associated with raters in this group was 0.3% which means rating behaviour did not substantially vary between raters in the occupational therapists’ group. About 18% of the observed variance was associated with interaction-error term (i.e., confounding factors).

Overall, the pattern of results was similar in the other two groups: the proportion of variance associated with the object of measurement (mobile app quality) was higher than the proportion of variance associated with raters or error term. However, the proportion of variance associated with raters in the older adult group was highest compared to other two groups. Since none of the apps used in this study were specifically relevant to needs of older adults, more variability in rater behaviour was expected compared to the mental health condition group.

### Construct Validity

**Figure 2.**
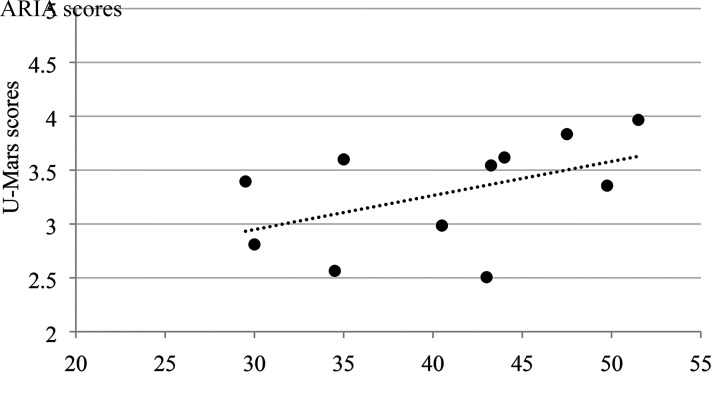
Correlation between ARIA and U-MARS (occupational therapists).

**Figure 3.**
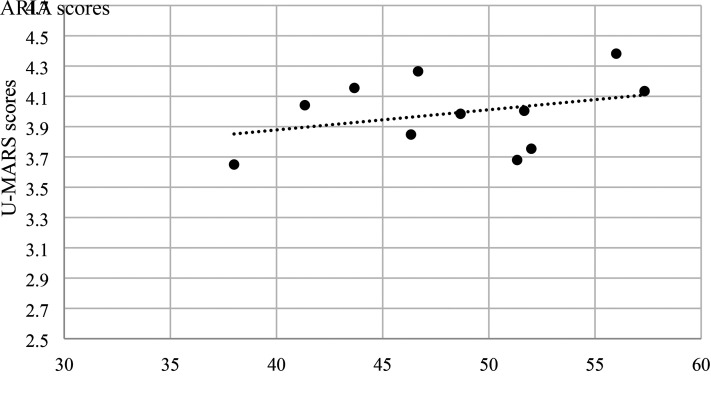
Correlation between ARIA and U-MARS (older adults).

**Figure 4.**
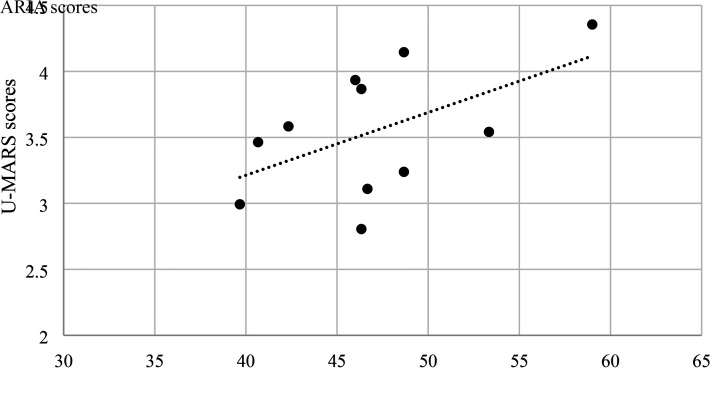
Correlation between ARIA and U-MARS (adults with mental health condition).

There was a low to moderate positive correlation between total scores of ARIA and U-MARS across all three groups of participants (Figures 2–4). The results suggest that ARIA and UMARS measure the same underlying construct (i.e., quality of apps) but the degree of overlap between their domains are minimal to moderate.

### Raters’ Comments

At the end of the study, each rater participated in an exit interview with the first author. Results of a thematic analysis indicated that raters found ARIA had items that were easier to understand, therefore made it easier to use compared to U-MARS; the new index covered more relevant criteria and showed them what to look for choosing a mhealth apps. Raters also reported that ARIA was more visually accessible as items were arranged on two pages, compared to five pages in U-MARS. Such features make ARIA a more feasible tool for clinical or community-based practice. The following quotes illustrate these themes:

Older adult 1: *“I think it [ARIA] has the right questions, and I think it covers all the essential points.”*

Older adult 2: *“The value of the [ARIA] was that it was short, and it just required you to add up things. The U-MARS was also relatively easy, but I needed to pull out the calculator from time to time, and sometimes it was four questions, sometimes three, and five and so the chances of error increased. it [ARIA] is easy to use.”*

Adult with mental 
health condition 2: *“The U-MARS was also good, but sometimes my math was probably off, I always had to go back and check if it was right.”*

Health care 
provider 1: *“Definitely yours [ARIA] is easier since it does not require me to calculate the averages, and it is easier to understand because you have just 5-point Likert scale and at the end, I just have to add the scores and write it down.”*

Health care 
provider 3: *“I liked your cues because being new at it not knowing where to find the information, not knowing what to look for, I really enjoyed having those cues to guide me to this is where you need to look for, so I thought those were really helpful.”*

## Discussion

In this study, we developed ARIA, a rating index to help the general public and health care providers such as occupational therapists to rate the quality of mobile health apps and select the ones that can best meet their needs. For this study, we tested our index with 10 users and 11 mental health apps. Among our participants, the occupational therapist group scored the quality of apps lower than the other groups. Closer analysis of their responses indicated that they were stricter with items relevant to trustworthiness, security, and privacy that resulted in lower total scores compared to other two groups. Such scrutiny can be due to an awareness of standards that occupational therapists follow in their professional practice regarding privacy of health information and evidence-based practice.

Overall, the occupational therapists were more consistent in their ratings compared to the other groups, confirmed by the low proportion of variance associated with raters. Each occupational therapist had rated apps considering the needs and requirements of the same mock patient. In contrast, raters in the two other groups rated apps based on their own needs, preferences, and expectations, all of which varied between individuals. The pattern of results in the adults with a mental health condition group was very similar to the occupational therapist group. For both groups, at least some of the apps used by the participants were relevant to their context. On the other hand, raters in the older adult group identified themselves as healthy. It is likely that not all the selected apps were relevant to their needs which could vary widely. This may have contributed to a higher proportion of variance associated with raters and consequently, a lower coefficient of reliability. A future study could examine apps that are relevant to older adults for a specific purpose, such as medication management, to see whether a similar pattern of variability emerges.

The reported analyses suggest that relevance of the app to the user is a pre-condition for deriving reliable and valid scores from ARIA. In other words, people will use ARIA to evaluate the quality of apps that are relevant to them. Occupational therapists responding on behalf of a client should know their clients’ needs and preferences well or alternatively, complete the tool with them. Additionally, factors such as perceived usefulness and perceived ease of use are subjective and may vary from one user to another. This has been shown in previous studies of technology acceptance and use (Venkatesh et al., 2012). Our findings were consistent with these studies by demonstrating the larger variance associated with older adult users, who differed in terms of technology savviness and needs compared to the other groups.

The inclusion of self-reported healthy older adults served an important purpose as they represent potential users of mental health apps for monitoring and prevention of mental health and other chronic conditions. For example, an app for medication management can be used by anyone such as an older adult who may have some age-related idiopathic memory lapse. Ratings by older adults informed us on how useful our index is to help older adults identify and rate issues related to trustworthiness, privacy policy, security measures, affordability, perceived ease of use, and functionality in a mobile app.

While currently there is no gold standard for rating the quality of mhealth apps, U-MARS is the only one developed for the public and widely used in app rating studies. A low to moderate positive correlation between the total scores of ARIA and U-MARS suggest that both tools measure a similar construct related to quality of apps. In addition, ARIA has some advantages compared to the existing app quality rating scales such as MARS and U-MARS. For example, the quality criteria measured by ARIA are supported by models of technology acceptance and usability (Supplemental Appendices 1 and 2). Further, the content of ARIA has been developed in an iterative process through multiple rounds of revisions in three phases by consumers of mobile health apps including older adults, patients, and health practitioners (Table 1). Finally, we developed ARIA considering the needs of both expert and novice consumers.

### Limitations

The sample size of this study was limited to 12 raters from whom 2 did not complete the study, and 11 apps. A future study would benefit from a larger sample of apps as well as raters. Additionally, this study was limited to raters from three groups of potential users of mobile health apps. Further studies with inclusion of more diverse healthcare providers or general users of mobile health apps are needed to determine the reliability of ARIA among other groups. Similarly, while the focus of this study was on mental health mobile apps, ARIA should be tested on apps related to other health care issues.

Another limitation of this study was that we selected apps from the Alberta Health Services Addiction and Mental Health Mobile Application Directory (2018). This decision was due to practical as well as ethical considerations. Since we included raters from the public, we could not risk exposing participants to any potentially harmful apps. Hence, we chose the apps from a directory that included apps that were curated and posted by a reputable public source or from trackable developers. Apps on the directory were similar to each other in many criteria. Nevertheless, the diversity of the scores by raters indicated that ARIA was capable of distinguishing different levels of quality among these apps.

This was a multimethod sequential study conducted in three phases. Although the phases of tool development (i.e., phases one and two) were not described in detail in this paper, they were not without limitations. In phase one, the focus groups were homogenous (i.e., all participants were from the same cohort). Additional focus groups with mixed cohorts could have resulted in more diverse exchange of opinions and richer data. In phase two, a Delphi method would have been more rigorous, however due to resource constraints, we employed a survey followed by a single focus group.

### Future Research

The results of this study provide a direction for future studies to evaluate the quality of mobile health apps. ARIA can be further validated on apps relevant to different health care conditions and by different consumers, including health practitioners in disciplines other than occupational therapy, and users with health conditions other than mental health. App developers and researchers can also use ARIA to develop mhealth apps to better address the needs of patients, health care providers, and family caregivers.

The results provide preliminary evidence for the validity of the ratings. Further studies with a focus on how the rating scores by this index may predict or impact acceptance and use of apps by different users are warranted to investigate the usefulness of the index scores.

One important step is to disseminate ARIA and make it accessible to as many users as possible. We are developing and validating an online (web-based) version of ARIA for the general public. The web-based version may record the ratings provided for each app and eventually create a crowd-sourced directory for mhealth apps. A French version of ARIA is also available, however this version has not yet been examined for reliability and validity.

## Conclusion

The number of mhealth apps is ever increasing. However, most are unregulated which creates risks for the public who may download apps that have questionable quality. Existing scales used to evaluate the quality of health apps are targeted at expert users. None of these scales are grounded in frameworks of usability assessment and quality evaluation of mobile apps. We developed the ARIA based on nine quality criteria from the relevant theories of usability and frameworks of app evaluation validated by potential users of mobile health apps. Our results suggest that occupational therapists, older adults, and adults with a mental health condition, may use ARIA to rate the quality of mhealth apps related to mental health conditions according to their goals, needs, and preferences.

## Key Messages

- The number of mhealth apps and their popularity among users, including healthcare providers and their clients are increasing.
- Current methods used to evaluate the quality of apps may not be suitable for users with limited experience with mobile health apps including older adults.
- Criteria based assessment tools such as the ARIA may educate healthcare providers and their clients about important factors to consider when selecting mobile health apps.

## Supplemental Material

sj-docx-1-cjo-10.1177_00084174221085451 - Supplemental material for Alberta Rating Index for Apps: Study of Reliability and Validity Click here for additional data file. Supplemental material, sj-docx-1-cjo-10.1177_00084174221085451 for Alberta Rating Index for Apps: Study of Reliability and Validity by Peyman Azad-Khaneghah, Mary Roduta Roberts and Lili Liu in Canadian Journal of Occupational Therapy

sj-docx-2-cjo-10.1177_00084174221085451 - Supplemental material for Alberta Rating Index for Apps: Study of Reliability and Validity Click here for additional data file. Supplemental material, sj-docx-2-cjo-10.1177_00084174221085451 for Alberta Rating Index for Apps: Study of Reliability and Validity by Peyman Azad-Khaneghah, Mary Roduta Roberts and Lili Liu in Canadian Journal of Occupational Therapy

sj-docx-3-cjo-10.1177_00084174221085451 - Supplemental material for Alberta Rating Index for Apps: Study of Reliability and Validity Click here for additional data file. Supplemental material, sj-docx-3-cjo-10.1177_00084174221085451 for Alberta Rating Index for Apps: Study of Reliability and Validity by Peyman Azad-Khaneghah, Mary Roduta Roberts and Lili Liu in Canadian Journal of Occupational Therapy
