# Supplementary material for: Alberta Rating Index for Apps: Study of Reliability and Validity
Source: Can J Occup Ther. 2022 Mar 16;89(3):326–38. doi: 10.1177/00084174221085451 (PMC9511245; doi:10.1177/00084174221085451)
Supplement: sj-docx-1-cjo-10.1177_00084174221085451 - Supplemental material for Alberta Rating Index for Apps: Study of Reliability and Validity [file sj-docx-1-cjo-10.1177_00084174221085451.docx]

Supplementary table 1.1

Models of usability used to develop << removed for blinding >>

| **Name of the model or framework** | UTAUT-2 | STAM | Nielsen Model |
| --- | --- | --- | --- |
| **Author(s)** | Venkatesh, Thong, and Xu (2012) | Renaud and Biljon  (2012) | Nielsen  (1993) |
| **Criteria** | 1) Performance Expectancy  2) Effort Expectancy  3) Social Influence  4) Facilitating conditions  5) Hedonic Motivation  6) Price Value  7) Habit | 1) Social influence  2) Perceived Usefulness  3) Facilitating Conditions  4) Ease of use and learning | 1) Usefulness   1. Utility 2. Usability 3. Easy to Learn 4. Efficient to Use 5. Easy to Remember 6. Few Errors   Subjectively pleasing  2) Social acceptability  3) Practical acceptability   1. Cost 2. Compatibility 3. Reliability |

Supplementary table 1. 2

Frameworks of app evaluation used to develop << removed for blinding >>

| **Name of the App Evaluation Framework** | App synopsis | Health Care Information and Management Systems Society (HIMSS) | Health on the net foundation | CIHR and MHCC Framework for Mental Health Apps |
| --- | --- | --- | --- | --- |
| **Author(s)** | Albrecht, Noll,  Von Jan, Jungnickel, and Pramann (2013) | Chan, Torous, Hinton, and Yellowlees (2015) | HON (2019) | CIHR and MHCC  (2018) |
| **Criteria** | 1) Imprint (information about manufacturer)  2) Rationale (app’s intended purpose) Rationale (app’s intended purpose)  3) Functionality  4)Validity and reliability of the information  5) Data requisitioning and management  6) Data protection  7) Data transmission | A) Usefulness:  1) Validity and accuracy (of app function)  2) Reliability (of app functions)  3) clinical Effectiveness  4) Time and number of sessions  B) Usability  1) Satisfaction  2) Usability (easy to use)  3) Disability accessibility  4) Cultural acceptability  5) Socio economic accessibility  C) Integration and Infrastructure  1) Security  2) Workflow  3) Data integration  4) Safety  5) Privacy | 1) Information is Authoritative (author is clear)  2)Purpose of the app is clear  3) Confidentiality  4) Information is referenced  5) Justification of claims  6) Developers contact details  7) Funding source is clear  8) Editorial content and advertisements are distinguished from one another | 1) Effectiveness  2) Clinical claims  3) Usability  4) User desirability  5) Security  6) Functionality  7) Interoperability  8) Supported platforms  9) Target users  10) Price  11) Transparency  12) Inclusion |
