# Supplementary material for: Alberta Rating Index for Apps: Study of Reliability and Validity
Source: Can J Occup Ther. 2022 Mar 16;89(3):326–38. doi: 10.1177/00084174221085451 (PMC9511245; doi:10.1177/00084174221085451)
Supplement: sj-docx-2-cjo-10.1177_00084174221085451 - Supplemental material for Alberta Rating Index for Apps: Study of Reliability and Validity [file sj-docx-2-cjo-10.1177_00084174221085451.docx]

# Alberta Rating Index for Apps (ARIA)-Care provider version

App Name: …………………………………………... I have used this app before: Yes □ No □

Use this index to rate the quality of a mobile health app for use by a client or patient (referred to as user). Please complete Part A and Part B.

| **Part A:**  **Complete this part before downloading the app on your phone or tablet.**   - First, **find the app on your online app store**. For Apple products open   “App store” for Android products go to the “Play Store.”   - Then, read the app description from the online app store and rate the extent to which you agree with the following statements. | | Strongly disagree | Disagree | Neutral | Agree | Strongly agree |
| --- | --- | --- | --- | --- | --- | --- |
| Purpose | 1. The description of the app’s purpose fits the user’s goals. **(You may find app description under “About this app” on Google Play; or “Preview” on iTunes)** | 0 | 1 | 2 | 3 | 4 |
| Trustworthiness | 2. Based on the description provided on the app store or the app developer’s website, the user can trust that relevant experts in the field have developed the app. | 0 | 1 | 2 | 3 | 4 |
|  | 3. The app description includes a statement about the risks associated with using the app. | 0 | 1 | 2 | 3 | 4 |
|  | 4. The app declares conflicts of interest, if any. | 0 | 1 | 2 | 3 | 4 |
| Privacy | 5. The app has a privacy policy that explains: (1) what information is collected by the app, (2) who will have access to this information, and (3) how this information will be used. **(Look for the “Privacy policy” of the app under**  **the app description on “App Store” for Apple products or “Play store” for Android products)** | 0 | 1 | 2 | 3 | 4 |
| Affordability | 6. The costs associated with using the app, including in-app purchases and subscription renewal fees, are affordable for the user. **(Go to the “App Store” for Apple products or “Play store” for Android products to learn how much does it cost to use the app)** | 0 | 1 | 2 | 3 | 4 |
| **Add up the scores for Part A** /24 | | | | | | |

1

| **Part B:** Use the app for at least 10 minutes or as long as you feel it is necessary to become familiar with its features. Try all **the links** and **buttons** on the screen. After you become familiar with the app, rate the extent to which you agree with the following statements. **You may go back to the app and check the features**. | | Strongly disagree | Disagree | Neutral | Agree | Strongly agree |
| --- | --- | --- | --- | --- | --- | --- |
| Security | 1. The app uses at least one security measure, such as **user name and password** or biometric identifiers **(fingerprints, face recognition)**, to  allow the user to access the app. | 0 | 1 | 2 | 3 | 4 |
|  | 2. The app asks for the user’s consent if it needs to access the phone's camera, microphone, user’s location, contacts, or photos. | 0 | 1 | 2 | 3 | 4 |
| Trustworthiness | 3. The app mentions the references for the health information that it provides. Examples of trustworthy references are scientific papers or websites of the governmental health organizations, universities, or not-for-profit health groups. | 0 | 1 | 2 | 3 | 4 |
| Ease of use | 4. Moving from one screen of the app to another would be easy for the user. | 0 | 1 | 2 | 3 | 4 |
|  | 5. It would be easy for the user to see components of the app such as text, icons, and buttons. **Pay attention to colours and sizes.** | 0 | 1 | 2 | 3 | 4 |
|  | 6. It would be easy for the user to understand the information provided by the app. **Pay attention to the text, graphs, tables, audio, or video.** | 0 | 1 | 2 | 3 | 4 |
| Functionality | 7. The app components work correctly. For example, it does not crash or all links work. | 0 | 1 | 2 | 3 | 4 |
|  | 8. The user would be able to customize the app settings **(language, font size, font colour, background colour, reminders, and notifications)** to her/his satisfaction. | 0 | 1 | 2 | 3 | 4 |
| Target users | 9. The content of the app is appropriate for the user considering her/his age, gender, education, and cultural background. | 0 | 1 | 2 | 3 | 4 |
| Usefulness and Satisfaction | 10. The app can help the user to achieve her/his goals. | 0 | 1 | 2 | 3 | 4 |
|  | 11. The user would find the app pleasing to use. | 0 | 1 | 2 | 3 | 4 |
|  | 12. The user would be satisfied with using the app. | 0 | 1 | 2 | 3 | 4 |
| **Add up the scores for Part B** /48 | | | | | | |
| Overall, I would recommend using this app to the user. | | 0 | 1 | 2 | 3 | 4 |
| Circle the number of stars that best represents your overall rating for quality of this app:  **(1 star =Worst app I have ever used; 5 Stars= Best app I have ever used)** | | 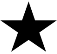 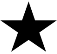 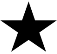 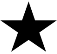 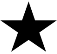 | | | | |

2

# Alberta Rating Index for Apps (ARIA)- User version

App Name: …………………………………………... I have used this app before: Yes □ No □

Use this index to rate the quality of a mobile health app. Please complete Part A and Part B.

| **Part A:**  **Complete this part before downloading the app on your phone or tablet.**   - First, **find the app on your online app store**. For Apple products open   “App store” for Android products go to the “Play Store.”   - Then, read the app description from the online app store and rate the extent to which you agree with the following statements. | | Strongly disagree | Disagree | Neutral | Agree | Strongly agree |
| --- | --- | --- | --- | --- | --- | --- |
| Purpose | 1. The description of the app’s purpose fits my goals. **(You may find app description under “About this app” on Google Play; or “Preview” on iTunes)** | 0 | 1 | 2 | 3 | 4 |
| Trustworthiness | 2. Based on the description provided on the app store or the app  developer’s website, I trust that relevant experts in the field have developed the app. | 0 | 1 | 2 | 3 | 4 |
|  | 3. The app description includes a statement about the risks associated with using the app. | 0 | 1 | 2 | 3 | 4 |
|  | 4. The app declares conflicts of interest, if any. | 0 | 1 | 2 | 3 | 4 |
| Privacy | 5. The app has a privacy policy that explains: (1) what information is collected by the app, (2) who will have access to this information, and (3) how this information will be used. **(Look for the “Privacy policy” of the app under the app description on “App Store” for**  **Apple products or “Play store” for Android products)** | 0 | 1 | 2 | 3 | 4 |
| Affordability | 6. The costs associated with using the app, including in-app purchases and subscription renewal fees, are affordable. **(Go to the “App Store” for Apple products or “Play store” for Android products to learn how much does it cost to use the app)** | 0 | 1 | 2 | 3 | 4 |
| **Add up the scores for Part A** /24 | | | | | | |

1

| **Part B:** Use the app for at least 10 minutes or as long as you feel it is necessary to become familiar with its features. Try all **the links** and **buttons** on the screen. After you become familiar with the app, rate the extent to which you agree with the following statements. **You may go back to the app and check the features**. | | Strongly disagree | Disagree | Neutral | Agree | Strongly agree |
| --- | --- | --- | --- | --- | --- | --- |
| Security | 1. The app uses at least one security measure, such as **user name and password** or biometric identifiers **(fingerprints, face**  **recognition)**, to allow users to access the app. | 0 | 1 | 2 | 3 | 4 |
|  | 2. The app asks for my consent if it needs to access the phone's camera, microphone, my location, my contacts, or my photos. | 0 | 1 | 2 | 3 | 4 |
| Trustworthiness | 3. The app mentions the references for the health information that it provides. Examples of trustworthy references are scientific papers or websites of the governmental health organizations, universities, or not-for-profit health groups. | 0 | 1 | 2 | 3 | 4 |
| Ease of use | 4. Moving from one screen of the app to another is easy for me. | 0 | 1 | 2 | 3 | 4 |
|  | 5. It is easy for me to see components of the app such as text, icons, and buttons. **Pay attention to colours and sizes.** | 0 | 1 | 2 | 3 | 4 |
|  | 6. It is easy for me to understand the information provided by the app. **Pay attention to the text, graphs, tables, audio, or video.** | 0 | 1 | 2 | 3 | 4 |
| Functionality | 7. The app components work correctly. For example, it does not crash or all links work. | 0 | 1 | 2 | 3 | 4 |
|  | 8. I can customize the app settings to my satisfaction **(Try to customize language, font size, font colour, background colour, reminders, and notifications).** | 0 | 1 | 2 | 3 | 4 |
| Target users | 9. The content of the app is appropriate for me considering my age, gender, education, and cultural background. | 0 | 1 | 2 | 3 | 4 |
| Usefulness and Satisfaction | 10. The app can help me to achieve my goals. | 0 | 1 | 2 | 3 | 4 |
|  | 11. The app is pleasing to use. | 0 | 1 | 2 | 3 | 4 |
|  | 12. I am satisfied with using the app. | 0 | 1 | 2 | 3 | 4 |
| **Add up the scores for Part B** /48 | | | | | | |
| Circle the number of stars that best represents your overall rating for quality of this app:  **(1 star =Worst app I have ever used; 5 Stars= Best app I have ever used)** | | 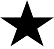 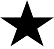 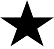 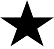 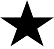 | | | | |

2

# Indice d’évaluation de la qualité des applications de santé mobiles (version pour les fournisseurs de soins)

Nom de l’application : …………………………………………... J’ai déjà utilisé cette application : Oui □ Non □ Utilisez ce tableau pour évaluer la qualité d’une application de santé mobile chez un client ou un patient (ci- après, « utilisateur »). Merci de remplir les Parties A et B.

| **Partie A:**  **Remplir cette partie avant de télécharger l’application sur votre téléphone ou votre tablette.**   - Tout d’abord, **trouvez l’application en ligne**. Pour les produits Apple, ouvrez « App store »; pour les produits Android, rendez-vous au « Play Store ». - Puis, lisez la description de l’application et indiquez dans quelle mesure   vous êtes d’accord (ou non) avec les énoncés suivants. | | Fortement en désaccord | Pas d’ accord | Neutre | D’ accord | Fortement d’ accord |
| --- | --- | --- | --- | --- | --- | --- |
| Objectif | 1. La description de l’objectif de l’application convient aux objectifs de  l’utilisateur. **(vous trouverez la description de l’application sous « About this app » *(Au sujet de cette app)* dans Google Play; ou sous « Preview » *(Aperçu)* dans iTunes)** | 0 | 1 | 2 | 3 | 4 |
| Fiabilité | 2. Selon la description fournie dans l’App Store ou sur le site Web du  développeur, l’utilisateur peut avoir confiance que l’application a été  développée par des experts compétents dans le domaine. | 0 | 1 | 2 | 3 | 4 |
|  | 3. La description de l’application comprend un énoncé portant sur les risques  associés à l’utilisation de l’application. | 0 | 1 | 2 | 3 | 4 |
|  | 4. Le cas échéant, les conflits d’intérêts sont énoncés. | 0 | 1 | 2 | 3 | 4 |
| Confidentialité | 5. La politique de confidentialité de l’application énonce : (1) les  renseignements recueillis par l’application; (2) qui aura accès à ces informations; et (3) comment ces informations seront utilisées. **(recherchez la politique de confidentialité dans la description de l’application dans**  **l’App Store pour les produits Apple ou dans le Play store pour les produits**  **Android)** | 0 | 1 | 2 | 3 | 4 |
| Abordabilité | 6. Les coûts associés à l’utilisation de l’application, y compris les achats intégrés  et les frais de renouvellement d’adhésion, sont abordables pour  l’utilisateur. **(rendez-vous dans l’App Store pour les produits Apple ou dans le Play store pour les produits Android pour savoir combien coûte l’utilisation de l’application)** | 0 | 1 | 2 | 3 | 4 |
| **Additionnez les scores de la Partie A** /24 | | | | | | |

1

| **Partie B :** Utilisez l’application pendant au moins 10 minutes, ou pendant aussi longtemps que vous l’estimez nécessaire pour vous familiariser à ses caractéristiques. Essayez tous **les liens** et **les boutons** sur l’écran. Après vous  être familiarisé avec l’application, évaluez la mesure dans laquelle vous êtes d’accord ou non avec les énoncés suivants. **Vous pouvez retourner à**  **l’application et vérifier ses caractéristiques**. | | Fortement en désaccord | Pas d’ accord | Neutre | D’ accord | Fortement  d’ accord |
| --- | --- | --- | --- | --- | --- | --- |
| Sécurité | 1. L’application est munie d’au moins une mesure de sécurité, comme un **nom d’utilisateur et un mot de passe,** ou encore des identificateurs biométriques **(empreintes digitales, reconnaissance faciale)** qui  permettent à l’utilisateur d’accéder à l’application. | 0 | 1 | 2 | 3 | 4 |
|  | 2. L’application demande le consentement de l’utilisateur si elle doit accéder à  sa caméra, son micro, son emplacement, ses contacts ou ses photos. | 0 | 1 | 2 | 3 | 4 |
| Fiabilité | 3. L’application indique les références des informations relatives à la santé qui y sont mentionnées. Les exemples de références fiables sont les articles scientifiques ou les sites Web des organismes de santé gouvernementaux,  les universités ou les groupes sans but lucratif œuvrant dans le domaine de la santé. | 0 | 1 | 2 | 3 | 4 |
| Facilité d’ utilisation | 4. L’utilisateur passera facilement d’un écran à l’autre de l’application. | 0 | 1 | 2 | 3 | 4 |
|  | 5. L’utilisateur verra facilement les éléments de l’application, comme le texte,  les icônes et les boutons. **Faites attention aux couleurs et aux tailles.** | 0 | 1 | 2 | 3 | 4 |
|  | 6. L’utilisateur comprendra facilement les informations fournies par  l’application. **Faites attention au texte, aux graphiques, aux tableaux, à**  **l’audio ou à la vidéo.** | 0 | 1 | 2 | 3 | 4 |
| Fonctionnalité | 7. Les éléments de l’application fonctionnent correctement. Par exemple, le système ne plante pas et tous les liens fonctionnent correctement. | 0 | 1 | 2 | 3 | 4 |
|  | 8. L’utilisateur pourra personnaliser les paramètres de l’application **(langue, taille et couleur de la police, couleur de l’arrière-plan, rappels et notifications)** à son goût. | 0 | 1 | 2 | 3 | 4 |
| Utilisateur s ciblés | 9. Le contenu de l’application est approprié pour l’utilisateur, compte tenu de  son âge, son sexe, sa formation et de ses antécédents culturels. | 0 | 1 | 2 | 3 | 4 |
| Utilité et satisfaction | 10. L’application peut aider l’utilisateur à atteindre ses objectifs. | 0 | 1 | 2 | 3 | 4 |
|  | 11. L’utilisateur trouvera l’application agréable à utiliser. | 0 | 1 | 2 | 3 | 4 |
|  | 12. L’utilisateur sera satisfait d’utiliser l’application. | 0 | 1 | 2 | 3 | 4 |
| **Additionnez les scores de la Partie B** /48 | | | | | | |
| En général, je recommanderais cette application à l’utilisateur. | | 0 | 1 | 2 | 3 | 4 |
| Entourez le nombre d’étoiles qui représente au mieux votre évaluation générale de la qualité de l’application :  **(1 étoile = La pire application que j’ai jamais utilisée; 5 étoiles = La meilleure application**  **que j’ai jamais utilisée)** | | 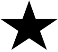 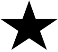 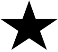 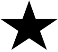 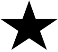 | | | | |

2

# Indice d’évaluation de la qualité des applications de santé mobiles (version pour les utilisateurs)

Nom de l’application J’ai déjà utilisé cette application : Oui □ Non □

Utilisez ce tableau pour évaluer la qualité d’une application de santé mobile. Merci de remplir les Parties A et B.

| **Partie A:**  **Remplir cette partie avant de télécharger l’application sur votre téléphone**  **ou votre tablette.**   - Tout d’abord, **trouvez l’application en ligne**. Pour les produits Apple, ouvrez « App store »; pour les produits Android, rendez-vous au « Play Store » - Puis, lisez la description de l’application et indiquez dans quelle mesure vous êtes d’accord (ou non) avec les énoncés suivants. | | Fortement en désaccord | Pas d’ accord | Neutre | D’ accord | Fortement d’ accord |
| --- | --- | --- | --- | --- | --- | --- |
| Objectif | 1. La description de l’objectif de l’application répond à mes objectifs. **(vous trouverez la description de l’application sous « About this app » (Au sujet de cette app) dans Google Play; ou sous « Preview » (Aperçu) dans iTunes)** | 0 | 1 | 2 | 3 | 4 |
| Fiabilité | 2. Selon la description fournie dans l’App Store ou sur le site Web du  développeur, j’ai confiance que l’application a été développée par des  experts compétents dans le domaine. | 0 | 1 | 2 | 3 | 4 |
|  | 3. La description de l’application comprend un énoncé portant sur les risques associés à l’utilisation de l’application. | 0 | 1 | 2 | 3 | 4 |
|  | 4. Le cas échéant, les conflits d’intérêts sont énoncés. | 0 | 1 | 2 | 3 | 4 |
| Confidentialité | 5. La politique de confidentialité de l’application énonce : (1) les  renseignements recueillis par l’application; (2) qui aura accès à ces informations; et (3) comment ces informations seront utilisées. (**recherchez la politique de confidentialité dans la description de l’application dans l’App Store pour les produits Apple ou dans le Play store pour les produits**  **Android)** | 0 | 1 | 2 | 3 | 4 |
| Abordabilité | 6. Les coûts associés à l’utilisation de l’application, y compris les achats intégrés et les frais de renouvellement d’adhésion, sont abordables. **(rendez-vous dans l’App Store pour les produits Apple ou dans le Play store pour les produits Android pour savoir combien coûte l’utilisation de l’application)** | 0 | 1 | 2 | 3 | 4 |
| **Additionnez les scores de la Partie A** /24 | | | | | | |

1

| **Partie B :** Utilisez l’application pendant au moins 10 minutes, ou pendant aussi longtemps que vous l’estimez nécessaire pour vous familiariser à ses caractéristiques. Essayez tous **les liens** et **les boutons** sur l’écran. Après vous être familiarisé avec l’application, évaluez la mesure dans laquelle vous êtes d’accord ou non avec les énoncés suivants. **Vous pouvez retourner à l’application et vérifier ses caractéristiques**. | | Fortement en désaccord | Pas d’ accord | Neutre | D’ accord | Fortement  d’ accord |
| --- | --- | --- | --- | --- | --- | --- |
| Sécurité | 1. L’application est munie d’au moins une mesure de sécurité, comme **un nom d’utilisateur et un mot de passe**, ou encore des identificateurs biométriques **(empreintes digitales, reconnaissance faciale)** qui  permettent aux utilisateurs d’accéder à l’application. | 0 | 1 | 2 | 3 | 4 |
|  | 2. L’application demande mon consentement si elle doit accéder à ma  caméra, mon micro, mon emplacement, mes contacts ou mes photos. | 0 | 1 | 2 | 3 | 4 |
| Fiabilité | 3. L’application indique les références des informations relatives à la santé qui y sont mentionnées. Les exemples de références fiables sont les articles scientifiques ou les sites Web des organismes de santé gouvernementaux, les universités ou les groupes sans but lucratif œuvrant dans le domaine de la santé. | 0 | 1 | 2 | 3 | 4 |
| Facilité d’ utilisation | 4. Je trouve qu’il est facile de passer d’un écran à l’autre de l’application. | 0 | 1 | 2 | 3 | 4 |
|  | 5. Je trouve qu’il est facile de voir les éléments de l’application, comme le  texte, les icônes et les boutons. **Faites attention aux couleurs et aux tailles.** | 0 | 1 | 2 | 3 | 4 |
|  | 6. Je trouve qu’il est facile de comprendre les informations fournies par  l’application. **Faites attention au texte, aux graphiques, aux**  **tableaux, à l’audio ou à la vidéo.** | 0 | 1 | 2 | 3 | 4 |
| Fonctionnalité | 7. Les éléments de l’application fonctionnent correctement. Par exemple,  le système ne plante pas et tous les liens fonctionnent correctement. | 0 | 1 | 2 | 3 | 4 |
|  | 8. Je peux personnaliser les paramètres de l’application **(essayez de personnaliser la langue, la taille et la couleur de la police, la couleur de l’arrière-plan, les rappels et les notifications)** à mon goût**).** | 0 | 1 | 2 | 3 | 4 |
| Utilisateur s ciblés | 9. Le contenu de l’application est approprié pour moi, compte tenu de  mon âge, mon sexe, ma formation et de mes antécédents culturels. | 0 | 1 | 2 | 3 | 4 |
| Utilité et satisfaction | 10. L’application peut m’aider à atteindre mes objectifs. | 0 | 1 | 2 | 3 | 4 |
|  | 11. L’application est agréable à utiliser. | 0 | 1 | 2 | 3 | 4 |
|  | 12. Je suis satisfait de l’utilisation de l’application. | 0 | 1 | 2 | 3 | 4 |
| **Additionnez les scores de la Partie B** /48 | | | | | | |
| Entourez le nombre d’étoiles qui représente au mieux votre évaluation générale de la qualité de  l’application: **(1 étoile = La pire application que j’ai jamais utilisée; 5 étoiles = La meilleure application que j’ai jamais utilisée)** | | 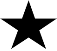 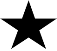 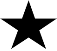 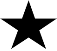 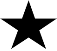 | | | | |

2
