# Supplementary material for: Alberta Rating Index for Apps: Study of Reliability and Validity
Source: Can J Occup Ther. 2022 Mar 16;89(3):326–38. doi: 10.1177/00084174221085451 (PMC9511245; doi:10.1177/00084174221085451)
Supplement: sj-docx-3-cjo-10.1177_00084174221085451 - Supplemental material for Alberta Rating Index for Apps: Study of Reliability and Validity [file sj-docx-3-cjo-10.1177_00084174221085451.docx]

**Appendix 3. Mock patient profile**

Gender: Male

Age: 65 years

Height: 170 Cm

Weight: 78 Kg (172 lb.)

Language(s) spoken English

Annual income: $20,000

Education: High school diploma

Occupation: Retired

Marital status: Single (Never Married)

Living arrangement: Lives alone

Housing: Lives in a subsidized condo

**Medical History**

Diagnosis: Bipolar mood disorder (Was diagnosed 40 years ago)

Chief complaint(s): Poor sleep, forgets to take medication on time, tired, and low energy during the day.

The patient is not a smoker, has no known substance abuse problems, and is not suffering from any eating disorders.

Medications: Mood stabilizers and Antidepressant-antipsychotic medication.

Psychotherapy: Cognitive behavioral therapy (CBT)

**Current Mental Status**

Suicidal ideation: No

Delusions/Hallucinations: No

Insight: Has insight into his condition,

Motivated to get better
